# Diagnostic Accuracy, Sensitivity and Specificity of Capillary POCT for Diagnosing Gestational Diabetes Mellitus

**DOI:** 10.3390/jcm15135070

**Published:** 2026-06-29

**Authors:** Lucie Wehling, Yvonne Heimann, Friederike Weschenfelder, Tanja Groten

**Affiliations:** 1 Department of Obstetrics, University Hospital Jena, Friedrich Schiller University, Am Klinikum 1, 07747 Jena, Germany; lucie.wehling@uni-jena.de (L.W.); yvonne.heimann@med.uni-jena.de (Y.H.); friederike.weschenfelder@med.uni-jena.de (F.W.); 2 Department of Obstetrics, University of Cologne, Faculty of Medicine and University Hospital Cologne, Kerpener Straße 34, 50931 Cologne, Germany

**Keywords:** gestational diabetes mellitus, 75 g oral glucose tolerance test, capillary point-of-care testing

## Abstract

**Background**: The 75 g oral glucose tolerance test (OGTT) is the gold standard for diagnosing gestational diabetes mellitus (GDM) and, according to current guidelines, is recommended for postpartum testing. German guidelines recommend a two-step screening approach, which can delay treatment initiation. In order to prevent complications for mother and child, treatment of GDM should be started as early as possible. To expedite clinical decisions, point-of-care testing (POCT) is often used alongside venous laboratory analysis. For historical reasons, capillary blood was used for POCT at our competence center. This analysis evaluates the diagnostic accuracy of capillary POCT compared to the venous reference standard analyzed in our department of clinical chemistry. **Methods**: We retrospectively analyzed 401 OGTTs (260 during pregnancy, 141 postpartum) with simultaneous capillary POCT and venous laboratory glucose measurements and investigated the agreement between the two methods. Optimal capillary cut-offs were determined using ROC analysis. **Results**: In pregnant women (*n* = 260), capillary POCT showed 80.8% sensitivity and 73.4% specificity. Regarding the diagnostic classification, the initial agreement with the reference standard at fasting was 71.9% (8.9% false positives, 19.2% false negatives). Optimized capillary cut-offs—fasting ≥95 mg/dL (5.3 mmol/L), 1-h ≥203 mg/dL (11.3 mmol/L), and 2-h ≥174 mg/dL (9.7 mmol/L)—increased the proportion of correctly classified cases to 85.5% (fasting), 97.0% (1-h), and 94.4% (2-h), respectively, and effectively eliminated false negatives. **Conclusions**: While capillary POCT offers >80% sensitivity, its false-positive rate of more than 20% must be managed. Utilizing optimized cut-off values can mitigate this uncertainty. If at least one of these cut-offs is exceeded, capillary POCT via StatStrip^®^ (Nova Biomedical Corporation, Waltham, MA, USA) provides a sufficient basis for treatment initiation.

## 1. Introduction

GDM is a glucose tolerance disorder first diagnosed during pregnancy. Globally, it affects one in six live births [1] and in Germany, its prevalence increased from 4.7% in 2013 to 8.5% in 2021 [2].

To mitigate the numerous short- and long-term complications for both mother and child [3], timely screening between the 24th and 28th weeks of pregnancy using the “one-step” 75 g OGTT or the “two-step” approach with a 50 g screen followed by a 75 g OGTT [4] is crucial for early diagnosis and therapy [5]. In the 75 g OGTT, GDM is diagnosed if at least one of the three established thresholds is reached or exceeded. According to the criteria of the International Association of Diabetes and Pregnancy Study Groups (IADPSG), these thresholds are: a fasting glucose value of ≥92 mg/dL (5.1 mmol/L), a one-hour value of ≥180 mg/dL (10.0 mmol/L), and a two-hour value of ≥153 mg/dL (8.5 mmol/L) [4]. For patients diagnosed with GDM, a 75 g OGTT is also recommended postpartum to screen for pre-existing metabolic disorders [4,6].

The S3-Level Guideline of the German Diabetes Association (DDG) and the German Society of Gynecology and Obstetrics (DGGG), as well as the IADPSG, refer to glucose measurement in venous plasma as the gold standard for diagnostic testing [4,7]. The guidelines, derived from the results of the study by Stahl [8], explicitly state that converting capillary plasma glucose values to venous plasma values is not permissible for diagnostic purposes. Despite its status as the gold standard, however, the central laboratory measurement presents several challenges, including preanalytical errors (e.g., sample handling) [4] and a significant delay in result communication and therapy initiation, often requiring additional consultations, especially in outpatient settings. This organizational deficiency can be addressed by POCT, which offers a patient-centered alternative that is both time-saving and enables immediate clinical decision-making and treatment initiation [9].

During a prospective clinical study, our group investigated different patient-centered diagnostic approaches to bridge the gap until lab results were available [10]. We compared six different methods, including laboratory measurement and POCT of capillary and venous blood, to diagnose GDM. The study found significant differences between all six glucose measurement methods used for diagnosing GDM, with no method being fully comparable to the reference method (venous blood and GlucoEXACT, SARSTEDT AG & Co. KG, Nümbrecht, Germany) [10]. Depending on the analytical technique, the detected GDM prevalence ranged widely (21.4–51.5%). POCT methods (HemoCue^®^ Hemocue AB, Ängelholm, Sweden and SuperGL easy+, Dr. Müller Gerätebau GmbH, Freital, Germany) using venous or capillary whole blood) showed substantial variability and poor agreement with the reference method [10]. Specifically, venous HemoCue^®^ markedly underestimated GDM prevalence, while capillary HemoCue^®^ and SuperGL easy+ detected similar overall prevalence but identified different women as having GDM [10]. Since, SuperGL easy+ using capillary blood performed best among POCT methods, we continued using this approach for POCT at our institution. Consequently, we selected Nova StatStrip^®^, which is approved for glucose measurement in capillary, venous and arterial blood [11], and implemented a unique dual-measurement protocol: Since 2016, all patients undergoing a 75 g OGTT have had their glucose levels simultaneously determined using the capillary StatStrip^®^ POCT device as the index test and the venous laboratory test as the reference standard (GlucoEXACT), while we still base definitive diagnosis on the venous gold standard.

The primary aim of this investigation is to evaluate the clinical safety of immediately utilizing capillary POCT results to commence essential GDM management steps (counseling, education and self-monitoring of blood glucose) before the final venous plasma result is reported. This requires determining the rate of agreement between the two methods and quantifying how frequently the initial POCT diagnosis would be revoked by the venous gold standard result. Furthermore, we seek to identify predictive factors among patient and test characteristics that can define subgroups where the capillary POCT result is sufficiently reliable to streamline the diagnostic pathway and allow for successful same-day GDM management.

## 2. Materials and Methods

### 2.1. Study Design

This study presents a retrospective analysis of 401 75 g OGTTs conducted at the Competence Center for Diabetes and Pregnancy at Jena University Hospital between 2016 and 2022. While 609 simultaneous glucose measurements (venous laboratory and capillary POCT) were initially recorded, 208 patients were excluded due to incomplete data. The resulting dataset included 260 diagnostic OGTTs in pregnant patients and 141 postpartum screenings in non-pregnant patients.

This study was designed as a retrospective secondary data analysis and received approval from the Ethics Committee of Friedrich Schiller University Jena (Ethics Number: 5280-09_17). In accordance with Section 27 of the Thuringian Hospital Act (ThürKHG), researchers are legally authorized to use clinical data collected during routine care for scientific purposes. Consequently, the anonymous use of this data is permitted without the requirement for explicit written consent.

### 2.2. Participants

Our study involved a series of pregnant and postpartum patients identified at our medical center. Eligible participants were identified between 2016 and 2022 based on two primary criteria: referral to our clinic following an abnormal 50 g glucose challenge test performed by outpatient gynecologists as part of routine GDM screening, or attendance for postpartum follow-up including a 75 g OGTT after a GDM-affected pregnancy. Since GDM is inherently sex-specific, the cohort consisted exclusively of female patients. Consequently, sex was not analyzed as a variable, and the findings are not directly generalizable to male populations.

### 2.3. Patient Data

Clinical data were extracted from electronic patient records. Gestational age was established via first-trimester crown-rump length measurement and calculated due date of 40 + 0 weeks. Maternal body mass index (BMI) was derived from height and recorded weight.

### 2.4. Test Methods

Before glucose testing, all patients had a fasting period of at least eight hours. Blood samples were collected at three time points during the OGTT: at baseline (fasting), at one hour, and at two hours.

Venous glucose was measured using the hexokinase method (Cobas^®^ 8000, Roche Diagnostics GmbH, Mannheim, Germany) in citrate-fluoride tubes (S-Monovette^®^ Gluco-EXACT, Sarstedt AG & Co. KG, Nümbrecht, Germany) to ensure immediate glycolysis inhibition. This reference standard was selected to minimize pre-analytical glucose loss, providing the most stable and accurate laboratory values [10] for comparison with the index test.

Capillary POCT and venous laboratory samples were collected simultaneously within a 5 min window. All POCT measurements were performed by trained staff in strict adherence to the quality assurance guidelines of the German Medical Association (Rili-BÄK) [12] and the accredited StatStrip^®^ system, which meets the criteria of ISO 15197 Standard [13]. Capillary blood was collected via fingerstick using a standardized protocol. After cleaning the site and allowing it to air dry, the first drop was discarded to prevent tissue fluid contamination. Blood was then collected via passive flow, strictly avoiding squeezing the puncture site.

The StatStrip^®^ system exhibits analytical precision with a coefficient of variation ranging from 8% at 50 mg/dL (2.8 mmol/L) to 4% at 600 mg/dL (33.3 mmol/L) [11].

The diagnostic cut-off values for both the index test (capillary POCT) and the reference standard (venous laboratory measurement) were pre-specified based on established guidelines. For the OGTT during pregnancy, a diagnosis of GDM was defined using the IADPSG criteria, as recommended by the S3-guidelines of the DDG and the DGGG. The thresholds for GDM were set at ≥92 mg/dL (5.1 mmol/L) for fasting glucose, ≥180 mg/dL (10.0 mmol/L) after one hour, and ≥153 mg/dL (8.5 mmol/L) after two hours.

Due to the limited number of cases involving impaired glucose tolerance (*n* = 4) and diabetes mellitus (*n* = 2) as determined by the venous reference standard in the postpartum subgroup, the agreement between both methods was evaluated exclusively for impaired fasting glucose (IFG). According to DDG criteria, IFG is defined as a fasting plasma glucose between 100 mg/dL (5.6 mmol/L) and 125 mg/dL (6.9 mmol/L). These pre-specified criteria were applied consistently to both the capillary POCT and the venous laboratory measurements to evaluate the diagnostic accuracy of the index test in a clinical setting.

The performers of both the index test and the reference standard were not blinded. Thus, clinical information and the respective test results were available to the performers during the evaluation of both measurement methods.

### 2.5. Primary Outcome

The primary objective of this study was to evaluate the diagnostic accuracy of capillary POCT compared to venous laboratory measurements to commence GDM management steps before the final venous plasma result is reported.

### 2.6. Statistical Methods

Estimates of diagnostic accuracy, including sensitivity, specificity, positive and negative predictive values, were calculated for both GDM and postpartum IFG screenings. Analytical agreement was assessed using Bland–Altman analysis to determine the bias and limits of agreement (LoA) between the two methods. For pregnant patients, this was done separately for the fasting, 1-h, and 2-h OGTT measurements.

**Figure 1:**
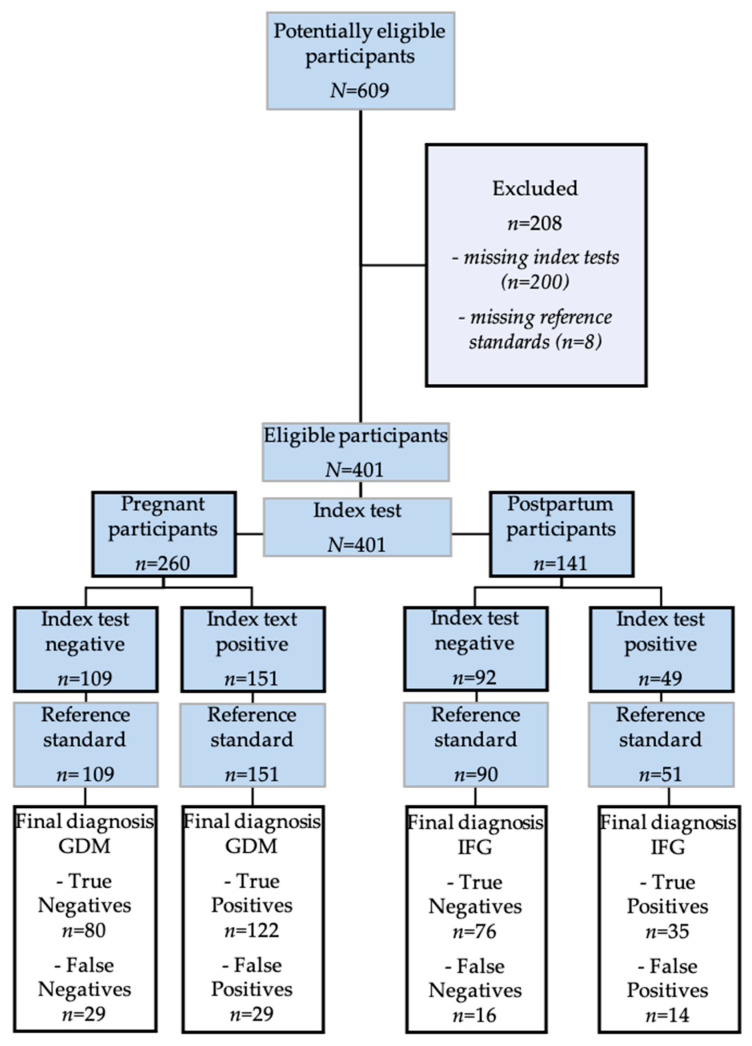
Diagram of participant flow through the study.

Regarding data integrity and missing data, patients were excluded from the study if any of the required OGTT time points (fasting, 1-h, or 2-h) were missing for either the index test or the reference standard. The flow of participants and the specific reasons for exclusion are detailed in the study flow chart (Figure 1). Indeterminate results, such as technical device errors, were treated as missing data and handled according to the same exclusion criteria.

The sample size was determined by the total number of eligible patients available at our clinic during the specified study period (convenience sample), representing the maximum cohort size achievable within the study’s timeframe.

To evaluate categorical variables, the Chi-square test was employed. Due to the non-normal distribution of the metric data, results are expressed as medians and interquartile ranges (IQR). Accordingly, the Mann-Whitney U test was utilized as a non-parametric method for comparing metric variables between groups. Statistical processing was performed using SPSS Statistics (Version 29.0, IBM Corporation, Armonk, NY, USA), with the significance level predefined at *p* < 0.05. To identify optimal capillary blood glucose thresholds during the OGTT, we utilized Youden’s Index (J = sensitivity + specificity − 1) as the primary criterion for cut-off selection. The calculation of this index was based on an underlying receiver operating characteristic (ROC) analysis with a 95% confidence interval to ensure statistical rigor.

## 3. Results

### 3.1. Description of Participants

The participant flow, from initial recruitment to final diagnostic categorization, is illustrated in Figure 1. Out of the 609 potentially eligible participants initially identified between 2016 and 2022, 208 individuals were excluded due to incomplete measurements in either the index test (StatStrip^®^ capillary POCT) or the reference standard (S-Monovette^®^ Gluco-EXACT venous laboratory measurement). This resulted in a final study cohort of 401 patients, all of whom underwent simultaneous capillary and venous glucose measurements. The cohort comprised 260 diagnostic OGTTs in pregnant women and 141 OGTTs conducted for postpartum screening, providing a definitive basis for the comparison of diagnostic accuracy.

Descriptive statistics for both groups are shown in Table 1.

### 3.2. Diagnostic Performance of Capillary POCT

The diagnostic performance of the index test compared to the reference is summarized in Table 2 and Table 3, which present the cross-tabulation of results for the pregnant and postpartum subgroups. Based on the venous gold standard, GDM was diagnosed in 151 of 260 patients, resulting in a prevalence of 58.1% in the pregnant subgroup. Similarly, IFG was diagnosed in 51 patients (36.2%) during the postpartum OGTT.

Sensitivity, specificity, and positive and negative predictive values were determined for the capillary POCT results, with the venous plasma laboratory results as reference. For pregnant patients (Table 4), capillary POCT results showed a sensitivity of 80.8% and a specificity of 73.4%.

Among the postpartum tests, capillary POCT measurement had a sensitivity of 68.6% and a specificity of 84.4% for the diagnosis of IFG (Table 5).

### 3.3. Group Comparison of Patient Characteristics

In the pregnant subgroup, both methods yielded concordant results in 202 of 260 cases (77.7%), showing either consistently positive or negative findings. In contrast, 58 patients showed discordant results, including 29 (11.15%) false-positive and 29 (11.15%) false-negative capillary POCT findings.

A comparison of the pregnant patient group with concordant results and the groups with either false-positive or false-negative capillary test results (Table 6) showed no significant difference in the clinical parameters examined.

For the diagnosis of IFG in postpartum individuals, the comparison between the venous gold standard and the capillary POCT method showed diagnostic concordance in 111 of 141 cases (78.7%). Capillary POCT resulted in 14 (9.9%) false-positive and 16 (11.4%) false-negative findings compared to the reference standard.

When comparing patient characteristics (Table 7), a significant difference (*p* = 0.002) was found in the patients’ body mass index (BMI) at the time of testing between the groups. Specifically, patients with concordant test methods had a median BMI of 26.9 kg/m^2^ (IQR, 24.3–31.8), which was significantly lower than the BMI of patients with false-positive capillary POCT results (36.4 kg/m^2^; IQR, 30.9–41.7). No further significant differences were found when comparing the concordant group to the group with false-negative capillary POCT results.

### 3.4. Methodological Agreement Between Capillary POCT and Venous Laboratory Results

#### 3.4.1. Bland–Altman Analysis

**Figure 2:**
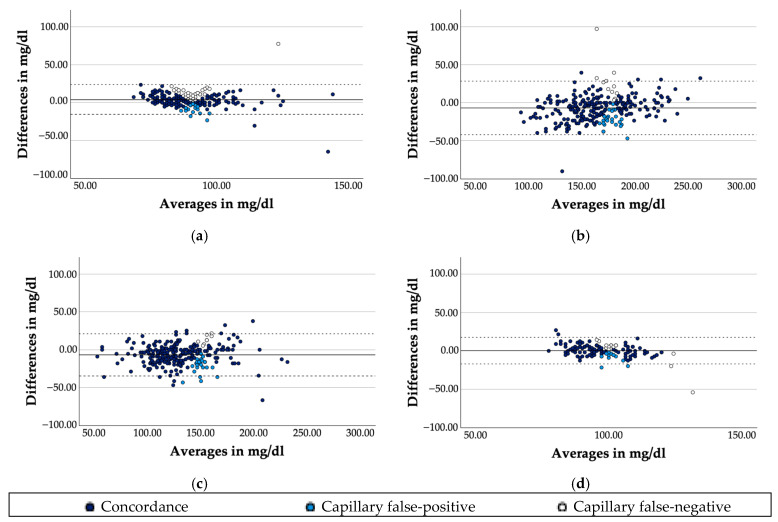
Bland–Altman plots: (**a**) Fasting measurement in pregnancy (GDM); (**b**) 1-h measurement in pregnancy (GDM); (**c**) 2-h measurement in pregnancy (GDM); (**d**) fasting measurement in the postpartum period (IFG).

Bland–Altman plots were used to assess overall agreement of capillary POCT and venous laboratory results (Figure 2).

Graphically, most values at all three time points fell within the LoA. The spread of mean differences was minimal at fasting measurement but increased at 1 h and 2 h time points, especially at the lower and upper ends of measurement range. The discordant results were predominantly centered within the middle range of measured values.

The mean difference between venous and capillary fasting measurements (Figure 2a) was 3.8 mg/dL (0.2 mmol/L) (95% confidence interval: 2.6 to 5.0 mg/dL (0.1 to 0.3 mmol/L)), indicating that the capillary POCT measured 3.8 mg/dL lower glucose concentrations than the venous laboratory method. The LoA were between 23.1 and −15.6 mg/dL (1.3 and −0.9 mmol/L). At 1-h (Figure 2b) and 2-h (Figure 2c) OGTT measurement, the mean differences shifted to −6.7 mg/dL (−0.37 mmol/L) (95% confidence interval: −8.9 to −4.6 mg/dL (−0.5 to −0.3 mmol/L)) and −6.8 mg/dL (−0.38 mmol/L) (95% confidence interval: −8.5 to −5.1 mg/dL (−0.5 to −0.3 mmol/L)), respectively. This shows that the capillary measured glucose concentrations were, on average, 6.7 mg/dL (0.37 mmol/L) and 6.8 mg/dL (0.38 mmol/L) higher than the venous measured glucose. The LoA were 28.5 to −42 mg/dL (1.6 to −2.3 mmol/L) at 1 h and 21.1 to −34.6 mg/dL (1.2 to −1.9 mmol/L) at 2 h.

In the Bland-Altman analysis for the fasting measurements of postpartum screening (Figure 2d), the mean difference was 0.3 mg/dL (0.02 mmol/L) (95% confidence interval: −1.1 to 1.8 mg/dL (−0.1 to 0.1 mmol/L)). The LoA ranged from 17.7 to −17 mg/dL (0.98 to −0.94 mmol/L). The data points showed an even distribution around the mean difference, with only a few outliers falling outside the LoA.

Sporadic outliers were identified across all time points. Longitudinal assessment showed no consistent patient-specific pattern, with no subject exhibiting recurrent deviations across consecutive measurements.

#### 3.4.2. Diagnostic Concordance Using Original Versus Optimized Thresholds

The Bland–Altman plots showed that discordant results occurred mainly in the middle measurement range. Therefore, we investigated whether the agreement and diagnostic certainty could be increased by determining new capillary cut-offs.

The diagnostic agreement for fasting measurements between the two methods was 71.9% (Table 8), with 8.9% false-positive and 19.2% false-negative capillary POCT results. A ROC analysis was performed to identify the range of discordant results, defining a measurement zone between a lower cut-off of 85 mg/dL (4.7 mmol/L) and an upper cut-off of 94 mg/dL (5.2 mmol/L). Outside of this defined range, the agreement was 87.2%, which included 5.4% false-positive and 7.4% false-negative capillary tests. Further analysis demonstrated that applying a single upper capillary cut-off of ≥95 mg/dL (5.3 mmol/L) resulted in an overall agreement of 85.5%, where the proportion of exclusively false-positive capillary POCT results was 14.5%.

The diagnostic agreement for the 1 h measurement between the capillary POCT and the venous gold standard was 85.8%. The capillary POCT showed a false-positive result in 10.0% of cases and a false-negative result in 4.2%. A measurement zone of discordant results was defined between a lower cut-off of 177 mg/dL (9.8 mmol/L) and an upper cut-off of 202 mg/dL (11.2 mmol/L). Outside of this zone, the agreement was 95.1%, comprising 0.5% false-positive and 4.4% false-negative capillary tests. Analysis further demonstrated that applying a single upper capillary cut-off of ≥203 mg/dL (11.3 mmol/L) resulted in an agreement of 97.0%, with 3.0% of the results being exclusively false-positive capillary tests.

The diagnostic agreement for the 2 h measurement was 87.3%. At this time point, the capillary POCT method yielded a false-positive result in 9.6% of patients and a false-negative result in 3.1%. A range where discordant results clustered was calculated with a lower cut-off of 150 mg/dL (8.3 mmol/L) and an upper cut-off of 173 mg/dL (9.6 mmol/L). Measurements outside of this range showed an agreement of 98.0%, with 1.0% each of false-positive and false-negative capillary tests. Subsequent analysis of this measurement range demonstrated that a single upper capillary cut-off of ≥174 mg/dL (9.7 mmol/L) resulted in an agreement of 94.4%, with 5.6% being exclusively false-positive capillary tests.

The overall diagnostic agreement of both methods throughout the entire OGTT was 77.7%, with 11.15% each of false-positive and false-negative POCT results. When the new capillary cut-offs were applied, the agreement increased to 91.5%. Among the POCT measurements that exceeded at least one of these derived cut-offs, 8.5% were found to be false-positive.

For postpartum OGTT as well, false-positive and false-negative POCT results were found to be clustered within the middle measurement range. Analysis using Youden’s index determined the optimal capillary cut-off to be ≥108 mg/dL (6.0 mmol/L). Concordance for the IFG category remained identical between the original and optimized capillary cut-offs (Table 9). Specifically, three of the 51 patients identified as IFG by the venous reference standard were classified as false negatives by the capillary method. These three individuals were categorized as having diabetes mellitus (≥126 mg/dL; ≥7.0 mmol/L) based on their capillary values. No patients with venous-confirmed IFG were classified as normoglycemic by the capillary method using the new thresholds.

## 4. Discussion

In this study, we compared capillary point-of-care testing with venous laboratory plasma glucose measurement as the reference standard for diagnosing gestational diabetes mellitus during pregnancy and impaired fasting glucose postpartum. Overall agreement between both methods was approximately 80% in both subgroups. When standard venous diagnostic thresholds were applied to capillary POCT, clinically relevant discordances were observed, including false-positive results in around 10% of cases and false-negative results in a similar proportion overall. Importantly, the optimized capillary-specific cut-offs identified in our study markedly improved diagnostic performance and highlight the strong clinical potential of capillary POCT. Their use may facilitate earlier initiation of time-critical treatment during pregnancy while potentially reducing the need for full postpartum OGTT testing in selected patients. These findings suggest that although capillary POCT should not be considered directly interchangeable with venous laboratory testing when identical thresholds are used, it may serve as a highly valuable and practical adjunct when method-specific cut-offs are applied.

The most relevant diagnostic limitation was observed for fasting glucose in pregnant women. At this time point, capillary POCT tended to underestimate venous plasma glucose, resulting in the lowest agreement and the highest false-negative rate. This is clinically important because elevated fasting glucose has been shown to correlate strongly with adverse pregnancy outcomes [14]. In contrast, agreement between methods was higher at the 1 h and 2 h OGTT measurements, although discordant results still occurred. Together, these findings demonstrate that the use of standard venous thresholds for capillary POCT is insufficient, particularly in the diagnostically sensitive fasting range.

Despite these limitations, our data show that diagnostic performance can be substantially improved through the use of capillary-specific thresholds. In our cohort, these capillary-specific cut-offs were identified as fasting ≥95 mg/dL (5.3 mmol/L), 1 h ≥203 mg/dL (11.3 mmol/L), and 2 h ≥174 mg/dL (9.7 mmol/L) during pregnancy, alongside a fasting threshold of ≥108 mg/dL (6.0 mmol/L) postpartum. While the use of a diagnostic range (utilizing lower and upper cut-offs) increased categorical agreement, it proved unsuitable as it failed to eliminate all false-negative results. In contrast, our newly defined thresholds markedly reduced false-positive classifications while ensuring that no false-negative diagnoses occurred. Although some false-positive results persist, potentially leading to premature counseling or therapy before venous confirmation, the rates remained within an acceptable clinical range, suggesting that the benefit of rapid therapeutic initiation outweighs the risk of transient over-diagnosis. Even in the postpartum subgroup, where IFG agreement appeared numerically static, these thresholds facilitated a critical diagnostic shift: discordant cases were categorized as diabetes mellitus rather than normoglycemic, thereby preserving clinical sensitivity. These results suggest that method-specific cut-offs are essential if capillary POCT is to be used safely in clinical practice.

The observed differences between capillary and venous glucose measurements are biologically plausible and analytically explainable. Under fasting conditions, arteriovenous glucose differences are minimal [15], whereas postprandial capillary glucose concentrations are physiologically higher than venous values [15,16]. This physiological pattern aligns with findings from Kuwa et al. and Larsson-Cohn, who both reported significant postprandial capillary–venous glucose differences, peaking at 1.4 mmol/L (at 60 min) [17] and 1.8 mmol/L (between 15 and 90 min) [18], respectively. In contrast, our study observed smaller but consistent mean differences of 0.37 mmol/L at 1 h and 0.38 mmol/L at 2 h.

Bland–Altman analyses demonstrated relatively small mean systematic bias but broad limits of agreement, indicating substantial individual variability. According to the criteria established by Bland and Altman, the clinical interchangeability of two methods depends on whether such discrepancies alter patient management [19]. In the context of GDM diagnosis, where decision thresholds are narrow, even small numerical deviations may lead to different diagnostic classifications. This likely explains why fasting glucose, despite small average bias, showed the poorest categorical agreement. Therefore, this individual variation in a vulnerable range necessitates the use of optimized cut-offs to ensure diagnostic safety and maintain high clinical concordance.

Reliable comparison with capillary POCT requires a robust venous reference method. The validity of venous glucose measurement depends heavily on preanalytical handling, particularly adequate inhibition of glycolysis [4,5,20]. We therefore used citrate-fluoride collection tubes, which have been shown to provide superior glucose stability compared with conventional sodium fluoride tubes [10]. This methodological approach strengthens the use of venous laboratory measurement as the diagnostic reference standard in the present study.

From a clinical perspective, capillary POCT offers several practical advantages. It is rapid, portable, less invasive, and simpler to perform [21] than repeated venous blood sampling. Immediate availability of results may facilitate same-visit counseling and early initiation of non-pharmacological treatment, including dietary and lifestyle interventions. Earlier patient education may also improve adherence and reduce the need for additional follow-up appointments. In our clinical setting, simultaneous capillary and venous sampling has enabled prompt management decisions while preserving diagnostic confirmation through laboratory analysis.

**Figure 3:**
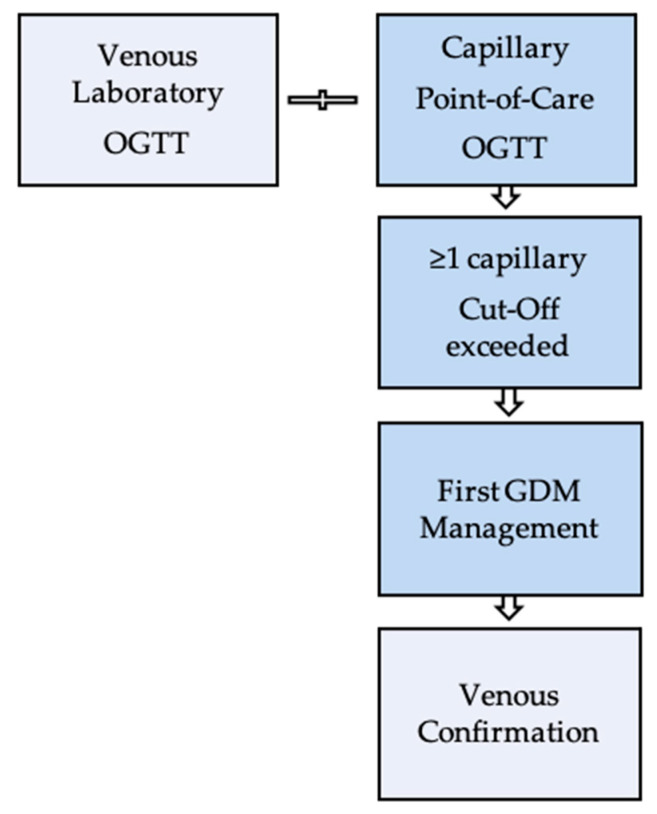
Diagram of clinical algorithm.

Based on our findings, we consider a combined diagnostic strategy to be currently the most appropriate approach for high-risk patients. If at least one optimized capillary threshold is exceeded, counseling and initial management can be started immediately while awaiting venous confirmation (Figure 3). Given the low residual false-positive rate after threshold optimization, the benefit of earlier treatment initiation likely outweighs the minor burden of an additional capillary puncture. However, sole reliance on capillary POCT without confirmatory venous testing requires further prospective validation before broad implementation.

We also explored potential factors influencing disagreement between methods. Hematocrit, a known source of error in some POCT systems [22,23], was not significantly associated with discordant results in our cohort. This lack of correlation likely reflects the built-in compensation algorithms of the device used [15,24]. Furthermore, evidence supports the high analytical performance of the StatStrip^®^ system, even in populations with hematocrit variations. For instance, Albloui and Lv demonstrated that, when using venous whole blood, the StatStrip^®^ system maintains consistent glucose measurements across varying hematocrit levels, showing no significant variation compared to laboratory-based reference methods [25,26]. Given that our cohort may exhibit altered hematocrit levels, this inherent technical robustness supports the reliability of our findings.

In the postpartum subgroup, higher BMI was associated with an increased likelihood of false-positive capillary results, suggesting that confirmatory venous testing may be particularly important in this population.

Several limitations should be acknowledged. First, this was a retrospective, single-center study conducted in a tertiary referral population of women with positive screening tests. This study design implies a selection bias, as the cohort consists exclusively of women who had already tested positive in a 50 g glucose challenge test, which explains the high GDM prevalence (58.1%) and limits the representativeness for the general obstetric population. Accordingly, external validity for low-risk screening populations is limited. Furthermore, the proposed capillary cut-offs were derived and evaluated within the same cohort. Thus, external validation in an independent population is required. Second, our analysis of potential confounders was limited. While we compared basic metabolic characteristics such as BMI and HbA1c between concordant and discordant cases, the influence of other factors such as insulin resistance and specific therapeutic interventions warrants further investigation. Third, the study design does not allow definitive separation of effects attributable to the testing method itself from those related to sample material (capillary versus venous blood). Future prospective multicenter studies should validate these optimized capillary thresholds in broader populations, assess the impact of these additional metabolic confounders, and evaluate whether capillary POCT alone can safely replace conventional OGTT pathways.

## 5. Conclusions

Capillary POCT using standard venous diagnostic thresholds shows insufficient sensitivity for reliable GDM diagnosis. However, application of optimized, time-point-specific capillary cut-offs substantially improves diagnostic performance, eliminating missed diagnoses while maintaining acceptable false-positive rates in this cohort. Although these results are promising, capillary POCT should currently be considered an adjunctive tool for rapid clinical decision-making rather than a replacement for the venous gold standard, pending further external validation in independent populations. Nevertheless, a strategy combining POCT with venous sampling offers a valuable balance between immediate counseling and diagnostic certainty, as exceeding optimized POCT thresholds may justify the initiation of non-pharmacological management while awaiting laboratory confirmation.

## Figures and Tables

**Table 1:** Baseline characteristics of the pregnant and postpartum individuals.

Parameter	Pregnancy (*n* = 260)		Postpartum (*n* = 141)	
	*n*	Median (IQR)	*n*	Median (IQR)
Age (years)	260	32.0 (29.0–35.0)	141	33.0 (30.0–37.0)
Week of pregnancy at the time of testing	260	26.0 (25.0–28.0)		
Prepartum BMI (kg/m^2^)	253	25.2 (22.5–29.5)		
BMI at time of testing (kg/m^2^)	239	28.71 (25.9–32.8)	99	28.0 (24.8–33.7)
Weight gain in pregnancy until test (kg)	233	8.2 (5.3–11.3)		
Test in summer (June–September)	260	92 (35.4%)	141	54 (38.3%)
HbA1c (%) at time of testing	229	5.0 (4.8–5.2)	123	5.5 (5.3–5.7)
HbA1c abnormal ^1^	229	13 (5.68%)	123	1 (0.8%)
Hematocrit (l/l)	253	0.35 (0.33–0.37)	122	0.40 (0.38–0.41)
Insulin therapy	247	50 (20.2%)		
Abnormal fasting glucose ^2^	260	116 (44.6%)	141	51 (36.2%)
Abnormal 1 h or 2 h glucose ^3^	260	95 (36.5%)	141	4 (2.8%)
Test abnormal with more than one value	260	70 (26.9%)	141	-
GDM (reference standard) ^4^	260	151 (58.1%)		
GDM (index test) ^5^	260	151 (58.1%)		
Impaired fasting glucose (reference standard) ^6^			141	51 (36.2%)
Impaired fasting glucose (index test) ^6^			141	49 (34.8%)
Impaired glucose tolerance ^7^			141	4 (2.8%)
Diabetes mellitus ^8^			141	2 (1.4%)

^1^ HbA1c in pregnancy ≥ 5.6%, postpartum HbA1c ≥ 6.5%. ^2^ Fasting glucose during pregnancy ≥ 92 mg/dL (5.1 mmol/L). ^3^ 1 h glucose ≥ 180 mg/dL (10.0 mmol/L) or 2 h glucose ≥ 153 mg/dL (8.5 mmol/L). ^4^ S-Monovette^®^ GlucoEXACT venous laboratory measurement. ^5^ StatStrip^®^ capillary POCT. ^6^ Fasting glucose postpartum 100–125 mg/dL (5.6–6.9 mmol/L). ^7^ 2 h glucose 140–199 mg/dL (7.8–11.0 mmol/L). ^8^ Fasting glucose ≥ 126 mg/dL (7.0 mmol/L) or OGTT 2 h value ≥ 200 mg/dL (11.1 mmol/L).

**Table 2:** Cross-tabulation of the index test results in the pregnant group.

Index Test Result	Reference Standard Positive	Reference Standard Negative	Total
Index test positive	122	29	151
Index test negative	29	80	109
Total	151	109	260

**Table 3:** Cross-tabulation of the index test results in the non-pregnant group.

Index Test Result	Reference Standard Positive	Reference Standard Negative	Total
Index test positive	35	14	49
Index test negative	16	76	92
Total	51	90	141

**Table 4:** Diagnostic accuracy of capillary POCT compared to venous laboratory method at OGTT during pregnancy.

	Estimate (%)	95% Confidence Interval
Sensitivity	80.8	0.74–0.87
Specificity	73.4	0.64–0.81
Neg. PV	73.4	0.64–0.81
Pos. PV	80.8	0.74–0.87

**Table 5:** Diagnostic accuracy of capillary POCT compared to venous laboratory method at postpartum OGTT.

	Estimate (%)	95% Confidence Interval
Sensitivity	68.6	0.54–0.81
Specificity	84.4	0.75–0.91
Neg. PV	82.6	0.73–0.90
Pos. PV	71.4	0.57–0.83

**Table 6:** Group comparison of concordant vs. discordant test results at OGTT during pregnancy.

	Concordant Results (*n* = 202)		Capillary False-Positive (*n* = 29)			Capillary False-Negative (*n* = 29)		
	*n*	Median (IQR)	*n*	Median (IQR)	*p*- Value	*n*	Median (IQR)	*p*- Value
Age (years)	202	32.0 (29.0–35.0)	29	30.0 (27.0–35.0)	0.228	29	31.0 (28.5–36.5)	0.804
Week of pregnancy	202	26.5 (25.0–28.0)	29	26.0 (25.0–28.0)	0.311	29	26.0 (25.0–28.0)	0.144
Prepartum BMI (kg/m^2^)	197	25.4 (22.4–29.8)	27	23.5 (21.9–25.3)	0.109	29	26.2 (22.9–32.0)	0.478
BMI at time of testing (kg/m^2^)	186	28.9 (25.6–32.9)	28	27.5 (26.3–30.6)	0.415	25	29.9 (26.5–34.4)	0.404
Weight gain until test (kg)	182	8.0 (4.9–11.1)	26	8.7 (6.0–12.4)	0.186	25	9.2 (6.5–10.9)	0.144
Test in summer (June–September)	202	73 (36.1)	29	8 (27.6)	0.412	29	11 (37.9)	0.839
HbA1c (%)	178	5.0 (4.8–5.3)	25	5.0 (4.8–5.2)	0.477	26	5.0 (4.8–5.2)	0.934
HbA1c abnormal ^1^	178	10 (5.6)	25	1 (4)	1.0	26	2 (7.7)	0.654
Hematocrit in (l/l)	196	0.35 (0.33–0.37)	29	0.35 (0.33–0.36)	0.423	28	0.35 (0.34–0.37)	0.217

^1^ HbA1c ≥ 5.6%.

**Table 7:** Group comparison of concordant vs. discordant test results at postpartum OGTT.

	Concordant Results (*n* = 111)		Capillary False-Positive (*n* = 14)			Capillary False-Negative (*n* = 16)		
	*n*	Median (IQR)	*n*	Median (IQR)	*p*- Value	*n*	Median (IQR)	*p*- Value
Age (years)	111	33.0 (30.0–37.0)	14	31.5 (31.0–38.0)	0.620	16	34.5 (30.5–37.0)	0.785
BMI at time of testing (kg/m^2^)	77	26.9 (24.3–31.8)	11	36.4 (30.9–41.7)	0.002	11	26.9 (25.2–29.1)	0.919
Test in summer (June–September)	111	46 (41.4)	14	4 (28.6)	0.402	16	4 (25)	0.278
HbA1c (%)	95	5.5 (5.3–5.6)	13	5.5 (5.3–5.7)	0.660	15	5.6 (5.2–5.7)	0.912
HbA1c abnormal ^1^	95	1(1.05)	13	-	-	15	-	-
Hematocrit (l/l)	94	0.40 (0.38–0.41)	13	0.39 (0.38–0.41)	0.749	15	0.39 (0.37–0.40)	0.127

^1^ HbA1c ≥ 6.5%.

**Table 8:** Diagnostic agreement of prepartum OGTT with capillary cut-offs.

Parameter	Concordant Results	Capillary False-Positive	Capillary False-Negative
Overall fasting values	187 (71.9%)	23 (8.9%)	50 (19.2%)
Capillary fasting value <95 mg/dL (5.3 mmol/L)	140 (68.3%)	15 (7.3%)	50 (24.4%)
Capillary fasting value ≥95 mg/dL (5.3 mmol/L)	47 (85.5%)	8 (14.5%)	-
Overall 1 h values	223 (85.8%)	26 (10.0%)	11 (4.2%)
Capillary 1 h value <203 mg/dL (11.3 mmol/L)	191 (84.1%)	25 (11.0%)	11 (4.9%)
Capillary 1 h value ≥203 mg/dL (11.3 mmol/L)	32 (97.0%)	1 (3.0%)	-
Overall 2 h values	227 (87.3%)	25 (9.6%)	8 (3.1%)
Capillary 2 h value <174 mg/dL (9.7 mmol/L)	210 (86.8%)	24 (9.9%)	8 (3.3%)
Capillary 2 h value ≥174 mg/dL (9.7 mmol/L)	17 (94.4%)	1 (5.6%)	-
Overall OGTT	202 (77.7%)	29 (11.15%)	29 (11.15%)
≥1 capillary cut-off exceeded	75 (91.5%)	7 (8.5%)	-

**Table 9:** Diagnostic agreement of postpartum OGTT with capillary cut-offs.

Parameter	Concordant Results	Capillary False-Positive	Capillary False-Negative
Overall fasting values	111 (78.7%)	14 (9.9%)	16 (11.4%)
Capillary fasting value <108 mg/dL (6.0 mmol/L)	89 (78.8%)	11 (9.7%)	13 (11.5%)
Capillary fasting value ≥108 mg/dL (6.0 mmol/L)	22 (78.6%)	3 (10.7%)	3 (10.7%)

## Data Availability

The datasets used and/or analyzed during the current study are available from the corresponding author on reasonable request.

## Abbreviations

The following abbreviations are used in this manuscript:

GDM	Gestational Diabetes Mellitus
OGTT	Oral Glucose Tolerance Test
POCT	Point-of-Care Testing
IADPSG	International Association of Diabetes and Pregnancy Study Groups
DDG	German Diabetes Association
DGGG	German Society of Gynecology and Obstetrics
BMI	Body Mass Index
LoA	Limits of Agreement
IQR	Interquartile Range
ROC	Receiver Operating Characteristic
